# The reality of task shifting in medicines management- a case study from Tanzania

**DOI:** 10.1186/s40545-015-0032-8

**Published:** 2015-04-06

**Authors:** Karin A Wiedenmayer, Ntuli Kapologwe, James Charles, Fiona Chilunda, Siana Mapunjo

**Affiliations:** Swiss Centre for International Health, Swiss Tropical and Public Health Institute, Basel, Switzerland; Regional Medical Office, P.O Box 320 Shinyanga, Shinyanga Region Tanzania; District Medical Office, P.O Box 1126 Chamwino, Dodoma Region Tanzania; Health Promotion and System Strengthening Project, P.O Box 29 Dodoma, Dodoma Region Tanzania; Ministry of Health and Social Welfare, Dar es Salaam, Tanzania

**Keywords:** Health workforce crisis, Pharmaceutical staff, Medicines management, Task shifting, Tanzania

## Abstract

**Objectives:**

Tanzania suffers a severe shortage of pharmaceutical staff. This negatively affects the provision of pharmaceutical services and access to medicines, particularly in rural areas. Task shifting has been proposed as a way to mitigate the impact of health worker shortfalls.The aim of this study was to understand the context and extent of task shifting in pharmaceutical management in Dodoma Region, Tanzania. We explored 1) the number of trained pharmaceutical staff as compared to clinical cadres managing medicines, 2) the national establishment for staffing levels, 3) job descriptions, 4) supply management training conducted and 5) availability of medicines and adherence to Good Storage Practice.

**Methods:**

A cross-sectional study was conducted in 270 public health facilities in 2011. A pre-tested questionnaire was administered to the person in charge of the facility to collect data on staff employed and their respective pharmaceutical tasks. Availability of 26 tracer medicines and adherence to Good Storage Practice guidelines was surveyed by direct observation. The national establishments for pharmaceutical staffing levels and job descriptions of facility cadres were analysed.

**Results:**

While required staffing levels in 1999 were 50, the region employed a total of only 14 pharmaceutical staff in 2011. Job descriptions revealed that, next to pharmaceutical staff, only nurses were required to provide dispensing services and adherence counselling. In 95.5% of studied health facilities medicines management was done by non-pharmaceutically trained cadres, predominantly medical attendants. The first training on supply management was provided in 2005 with no refresher training thereafter. Mean availability of tracer medicines was 53%, while 56% of health facilities fully met criteria of Good Storage Practice.

**Conclusion:**

Task shifting is a reality in the pharmaceutical sector in Tanzania and it occurs mainly as a coping mechanism rather than a formal response to the workforce crisis. In Dodoma Region, pharmacy-related tasks and supply management have informally been shifted to clinical staff without policy guidance, explicit job descriptions, and without the necessary support through training. Implicit task shifting should be recognized and formalized. Job orientation, training and operational procedures may be useful to support non-pharmaceutical health workers to effectively manage medicine supply.

## Introduction

The serious shortage of trained health workers globally has been identified as one of the most critical constraints to achieving health and development goals [1]. The workforce crisis is aggravated by an uneven distribution of the workers within countries and a generally inadequate level of staffing in rural and remote areas. Health workers are often poorly trained and under-paid, insufficiently supported and equipped, and consequently unmotivated. In addition to the low numbers of health workers providing health care, the quality of services is affected in situations of staff shortage which discourages patients’ confidence in the health system. This combination of poor quality of care and limited coverage of health care services inevitably translates into poor health indicators and outcomes [2-4].

Much like most low-income countries, Tanzania is suffering a critical crisis in human resource for health [5]. The country also faces a severe shortage of pharmaceutical staff, specifically. This has implications for inequalities in access to medicines and medicines expertise in general.

The number of pharmacists per 10,000 population worldwide varies considerably between countries and generally correlates with economic development indicators at country level, ranging from 0.02 in Somalia to 25.07 in Malta. The mean number for all countries is 6.02, while the African region has the most intense pharmacy workforce crisis, with an average of only 0.55 pharmacists per 10,000 population [6]. In 2009, the pharmaceutical human resources report of Tanzania [7] identified a total of 640 pharmacists, 479 pharmacy technicians, and 376 pharmacy assistants with a mean density of pharmacists of only 0.18 per 10,000 population nationally. In addition to a critical shortage of all categories of pharmaceutical workforce, these findings point to a skills mix imbalance with a large number of highly qualified staff (pharmacists) relative to the low number of staff with basic training (pharmaceutical technicians and pharmacy assistants).

Pharmacists in Tanzania tend to mainly work in urban areas and at higher levels of the health system. Vacancies at lower levels and in more rural regions are most often filled by other cadres without pharmaceutical competencies. This imbalance poses a major challenge to the provision of pharmaceutical services, particularly in rural areas where the majority of the population lives [6].

The impact of the pharmacy staff interventions on health services and outcomes has been described in some studies providing evidence of improved medication use, patient outcomes [8], improved adherence [9] and cost savings [10]. To ensure that medicines are made available to the population, functioning pharmaceutical regulatory and supply systems are required, with adequate numbers of qualified pharmaceutical personnel [11]. Expected roles of pharmaceutical staff at service delivery level include ensuring uninterrupted supply of quality medicines, adequate management and responsible use of these medicines. In a context of staff shortage, the question arises whether the supply of medicines can only be managed effectively by pharmaceutical staff. Or could any duly trained cadre perform the same activities with similar results? The concept of task shifting needs to be explored.

There is a large body of literature on access to medicines and various interventions to improve access, yet there has been little improvement in recent years in the availability of essential medicines in developing countries [12,13]. International efforts to improve access to essential medicines focus mainly on supply chain strengthening, price reductions and efforts to increase Research and Development (R&D) [13]. Effective supply management contributes to well-stocked pharmacies, encourages the community to seek and use health services and promotes enrolment in health insurance schemes [14-16].

A recent study explored access to medicines from a health system perspective, analysing existing access frameworks [17]. While human resources are one of the building blocks of health systems, they are only mentioned by the World Health Organization (WHO) with reference to prescribing and dispensing of medicines as a core activity of health workers.

Task shifting has been proposed as one of the ways to mitigate the impact of health worker shortfalls [18]; and as an opportunity for countries to build equitable and sustainable health systems [18]. Task shifting is defined by WHO as “the rational redistribution of tasks among health workforce teams”, with specific tasks moved from highly qualified health workers to health workers with shorter training and fewer qualifications in order to make efficient use of the available human resources [19]. It is not a new concept and is common in many high-income countries in which lower-level cadres with appropriate training and supervision perform tasks delegated or shifted to them from higher-level cadres. There is a growing body of literature supporting the use of task shifting in middle-income and low-income countries, mainly addressing specific health conditions such as HIV, TB or mental health [20,21]. Various scenarios of task shifting have been described, such as delegating tasks to professionals with less training, for instance from a nurse to a community health worker, creating a new cadre or delegating tasks to non-professionals [22]. An example at lower levels of the health system is the dispensing of medicines by nurses due to lack of pharmacists [23]. However, there are challenges with task shifting and results have not always been favourable. In a study on task shifting in HIV/AIDS, quality and safety concerns, professional and institutional resistance, and the need to sustain motivation and performance were noted [24].

The current study was conducted on behalf of the Health Promotion and System Strengthening Project (HPSS) which aims to improve the health of the population in Dodoma Region, Tanzania. The purpose of this study was to understand the context and extent of task shifting in pharmaceutical management in Dodoma Region. Specifically, we explored the number of trained pharmaceutical staff as compared to clinical cadres managing medicines supply in public health facilities of Dodoma Region, job descriptions, pharmacy staffing levels in comparison with the Ministry of Health guidelines for health facilities and training conducted in supply management. Finally, we assessed availability of tracer medicines and adherence to Good Storage Practice (GSP) guidelines – two key indicators of a medicines supply chain.

## Methods

The health facility study was done in the six districts of Dodoma Region, Tanzania. The public health system comprises a pyramidal network of dispensaries, health centres and hospitals. The dispensary is the most peripheral level of service delivery, catering for between 6,000 to 10,000 people. Health centres serve about 50,000 people providing in-patient services for patients referred from lower levels. District hospitals provide services to an average of 250,000 people. The regional hospital provides additional services not available at district hospitals and has specialists in various medical fields [25].

A cross-sectional study was conducted in 270 public health facilities of Dodoma Region, Tanzania in 2011 to explore staffing level of health facilities and their cadres responsible for medicines management. A questionnaire was used to document information and observations on the number and cadre of health workers, their role in medicines management, availability of tracer medicines and standard indicators of GSP. Main interviewees were facility in-charges. Pharmaceutical staff in Tanzania is categorised based on degree and training into *pharmacist* with a bachelor of pharmacy degree (BPharm) after 4 years of training, *pharmacy technician* with a diploma in pharmaceutical sciences after 3 years of training and *pharmacy assistant* with a certificate in pharmaceutical sciences after 2 years of training. A list of 26 tracer medicines was developed based on the National Essential Medicines List (NEML) and focusing on prevalent priority diseases in Dodoma Region. Availability of a medicine was defined as being in stock on the day of the study. The questionnaire was pre-tested, amended and questions were coded to allow data processing and analysis. GSP was defined in a checklist and standardized with 15 parameters including storage space, equipment and order, storage conditions regarding temperature, humidity, pest control and security, availability of cold storage, and handling of medicines. GSPwas considered satisfactory when all 15 criteria were met. Assessment was done by observation of storage conditions. Four experienced Tanzanian pharmacists were trained for the data collection.

The national establishment for staffing levels and allocation of pharmaceutical staff in the public sector of 1999 [26] valid at the time of the study was analysed by the investigators by using the recommended figures per level of health facility and calculating expected levels in fully staffed health facilities of Dodoma Region. A revised and updated staff establishment was published in early 2014 with significantly increased requirements for the number of pharmaceutical staff per level of care [27]. These new requirements are more in line with the observed need of sufficient and qualified health workers for appropriate quality of care. For this reason, the revised national establishment of 2014 was also analysed by the investigators.

National job descriptions [28] of all concerned cadres were analysed by investigators using the criteria and standards for good pharmacy practice (GPP) established by the International Pharmaceutical Federation (FIP), organised around major roles for pharmacy staff. These were grouped into medicines supply management (prepare, obtain, store, secure, distribute, administer, dispense and dispose of medical products) and medication therapy management (provide effective medication therapy management) [29].

All 270 health facilities of Dodoma Region (sampling frame) were included and surveyed. Data was collected using conventional paper forms. A statistician managed data processing including data entry, cleaning, and analysis. Data were analysed using SPSS (version 12). Ethical clearance was granted by the University of Dodoma. A letter of introduction from each Council Health Management Team, signed by the District Medical Officer and the District Executive Director, was presented to each facility and verbal consent was sought before proceeding with interviews.

## Results

In 2011, Dodoma Region comprised 6 districts and a total of 270 health facilities: 1 regional hospital, 6 district hospitals, 27 health centres and 236 dispensaries. Of 270 health facilities, 247 (91.5%) were open and staffed at the time of the survey. 19 health facilities were closed, while no staff was present in 4 facilities.

### Staff managing medicines

Of the 247 facilities surveyed (Table 1), only 5 health facilities, notably hospitals, employed a trained pharmacist, 7 health facilities had a pharmacy technician and 2 health facilities had a pharmacy assistant. In 236 of 247 open and staffed facilities (95.5%), medicines management was done by non-pharmaceutically trained cadres comprising nurses, medical attendants or clinical officers. The relative percentage of non-pharmaceutically trained staff managing medicines is summarized in Table 1 below.

**Table 1:** Staff managing medicines at health facility (N = 247)*

**Cadre**	**Number**	**Percent %**
Pharmacist	5	2.0
Pharmacy technician	7	2.8
Pharmacy assistant	2	0.8
**Total pharmaceutical staff**	**14**	**5.6**
Clinical officer	72	29.1
Medical attendant	108	43.7
Nurse	56	22.7
**Total non-pharmaceutical staff**	**236**	**95.5**

* 3 pharmaceutical staff worked at the regional hospital leading to 3 more non-pharmaceutical staff managing medicines in dispensaries.

Pharmaceutical staff was stratified according to type and level of health facility. District and regional hospitals employed most of the recommended pharmaceutical personnel; health centres employed few, while dispensaries employed none. None of the 213 dispensaries and only four of the 27 health centres employed a pharmaceutically trained staff. The distribution of pharmaceutical staff by level of care is shown in Table 2 below.

**Table 2:** Number of pharmaceutical staff by type of functional facility*

**Type of facility**	**Number of health facility**	**Number of pharmaceutical staff**
Dispensary	213	0
Health centre	27	4
District/regional hospital	7	10
**All**	**247**	**14**

*open and staffed.

Job descriptions by the Ministry of Health and Social Welfare (MoHSW) were analysed with regard to activities related to medicines supply management for various non-pharmaceutical cadres [28]. None of the clinical cadres present in the surveyed health facilities in Dodoma Region had any activity listed concerning duties in supply or medication management (Table 3). Only nurses are required to provide dispensing and adherence counselling.

**Table 3:** **Pharmacy-related duties per cadre in national job descriptions (MoHSW)**

**Cadre**	**Supply management duties**	**Medication therapy management duties**
Medical Doctor	N/A	N/A
Assistant Medical Officer	N/A	N/A
Clinical Officer	N/A	N/A
Medical attendant	N/A	N/A
Nurse	Dispensing	Adherence counselling

### Required numbers of pharmaceutical staff

According to the 1999 requirements, the required number of pharmaceutical staff for all 270 health facilities, based on the number of facilities and their level of care, would theoretically amount to a total of 50 workers. In reality, the region employed only a total of 14 pharmaceutical staff at the time of the study.

The national establishment for recommended pharmaceutical staffing levels at public health facilities of 1999 [26] valid at the time of the study was recently revised with minimum and maximum numbers of required health workers per level of care [27]. Based on the new establishment of staffing levels in 2014, the requirement would be 303-398 pharmaceutical health workers for the whole region [28]. Table 4 summarizes national facility staffing requirements in 1999 and 2014.

**Table 4:** **Establishment for facility allocation of pharmaceutical staff (MoHSW)**

**Level of duty resp. health facility (No)**	**MoHSW establishment**	**Pharmacist**	**Pharmaceutical technician**	**Pharmacy assistant**	**Total per facility 1999**	Total per facility 2014*
Region (1)	1999	1	0	0	1	
	**2014**	**1**	**0**	**0**		**1**
District (6)	1999	1	1	0	2	
	**2014**	**1**	**0**	**0**		**1**
Regional hospital (1)	1999	2	0	2	4	
	**2014**	**1-4**	**3-5**	**5-14**		**9-23**
District hospital (6)	1999	0	1	1	2	
	**2014**	**1-2**	**2-3**	**1-8**		**4-13**
Health Centre (27)	1999	0	0	1	1	
	**2014**	**0**	**1**	**0-1**		**1-2**
Dispensary (236)	1999	0	0	0	0	
	**2014**	**0**	**0**	**1**		**1**

*Numbers in minimum and maximum.

### Training on supply management

In Dodoma Region, the first and only training on supply management with the national Integrated Logistic System (ILS) was provided in 2005, with no refresher training until 2012, after conclusion of the survey. Both trainings were provided to the in-charge of a health facility. However, the actual work is often done by medical attendants and nurses. There is no formal induction or orientation for new staff at district level.

### Availability of medicines

Mean availability of 26 tracer medicines in all health facilities of Dodoma Region was 53% with significant variability between types of health facility. Corresponding mean stock-out rates in all facilities were in the order of 47%. Hospitals had higher availability of tracer medicines. Disaggregated by type of facility, this difference may have been driven by the presence of pharmacists in hospitals (Table 5).

**Table 5:** **Availability of tracer medicines and adherence to Good Storage Practice guidelines by type of facility**

**Type of facility**	**N**	**Mean availability in %**	**All GSP criteria met in %**	**Number of pharmaceutical staff**
Dispensary	213	52	50	0
Health centre	27	56	63	4
District/regional hospital	7	77	56	10
**All**	**247**	**53**	**56**	**14**

### Good storage practice

GSP is defined and standardized by a number of parameters. 56% of all health facilities had all conditions fulfilled for GSP with little difference observed between hospitals, health centres and dispensaries, despite more pharmaceutical staff being available in hospitals. In other words, storage practice is equally well or poorly done by non-pharmaceutically trained staff and by qualified pharmaceutical staff (Table 5).

## Discussion

### Pharmaceutical workforce shortage

**Figure 1:**
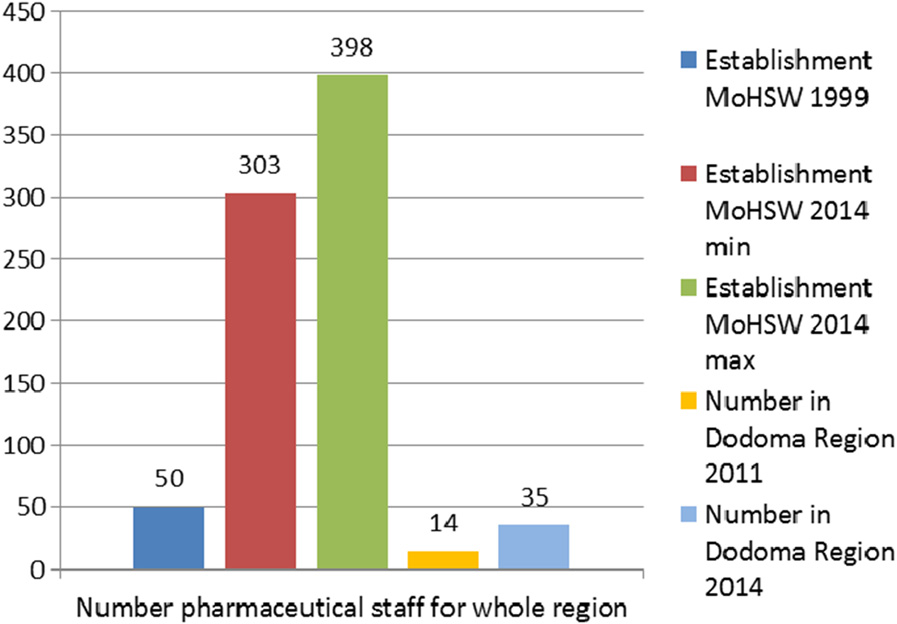
**MoHSW establishments of facility staffing levels 1999 resp. 2014 and actual numbers of pharmaceutical staff for Dodoma Region.**

In 95.5% of the public health facilities of Dodoma Region assessed by the study, medicines management is handled by medical attendants, clinical officers or nurses whose main task is clinical care of patients. These findings confirm the critical need for trained pharmaceutical staff. The pharmaceutical human resources report of Tanzania [7] revealed a total number of pharmaceutical personnel (1495) which is insufficient to staff the 5241 health facilities and pharmaceutical outlets in the country. The report showed that pharmaceutical services are provided by unqualified (non-pharmaceutical) personnel in over 70% of the facilities. Not surprisingly, vacancy rates are high in the Tanzanian public sector with 55% vacancy rate for pharmacists, 56% for pharmaceutical technicians and 77% for pharmacy assistants [30]. The national establishment for pharmaceutical staff allocation from 1999 was outdated at the time of the study and may have been based on available pharmacy staff in Tanzania at the time. Clearly, it did not reflect the realities and needs in the field in 2011. A revised and updated staff establishment was published in early 2014 describing significantly increased requirements for the number of pharmaceutical staff per level of care [27]. Based on this new guideline, the requirement in 2014 would be a minimum of 303 and a maximum of 398 pharmaceutical personnel for the whole region. This is in stark contrast to the actual numbers of a total of 14 pharmaceutical staff at the time of the study. The new requirements indicate the understood need of sufficient and qualified health workers for appropriate quality of care. Raising the targets is a first step towards initiating measures to increase the health workforce, given the dire reality of the workforce shortage in Tanzania. In early 2014, an internal survey by the Dodoma regional pharmacist recorded a total number of pharmaceutical staff of 35 in the region, documenting a modest increase in pharmaceutical staffing levels [31] Figure 1.

Discussions and interventions relating to the relative proportions of various pharmacy cadres (pharmacist, pharmaceutical technician and pharmacy assistant) are urgently needed. The pharmacy staffing pyramid in Tanzania is inversed with a high number of qualified pharmacists relative to the low number of staff with basic training such as technicians and assistants [32]. This negatively affects pharmaceutical service provision at lower level health facilities such as dispensaries, the large majority of all facilities, where pharmacy assistants are most likely to be posted. Pharmacists on the other hand would be ill-placed in a rural dispensary considering their long and costly education, as well as the waste of knowledge and skills which would not be fully utilized at this level of the health system. Rather, a lower cadre trained in a modular educational structure would be required and would have better chances of retention in rural areas. For example, a one year training for dispensers who could be upgraded to pharmacy assistant might mitigate the dire need for pharmaceutically trained health workers in primary health care facilities.

The analysis of job descriptions revealed that, on paper, the non-pharmaceutical cadres staffing health facilities in Tanzania such as clinical officers, medical attendants and nurses are not required to perform activities in medicine supply chain management. The only required involvement specified in medicine supply concerns dispensing and adherence counselling by nurses. In reality however, the person in charge of a health facility, theoretically a clinical officer, is usually given the responsibility to manage medicines supply. In dispensaries with general staff shortage, the person in charge - and therefore managing supply activities - may be a nurse or medical attendant. In facilities with sufficient clinical staff but no pharmaceutical personnel, the nurse will be responsible for stock management while forecasting and ordering will be done together with the in-charge of the facility.

### Training on medicine supply management

As medicine supply management is not formally part of clinical staff duties, knowledge and skills have to be acquired on the job. The only comprehensive training on supply management organized by the MoHSW in 2005 [33] was provided to the person in charge of a health facility while the actual work is usually done by the facility medical attendant or nurse. Transfer of knowledge to other staff is informal and usually dependent on the motivation of the in-charge of a facility. Newly recruited staff are not formally oriented and trained in supply management. After the one-time training in the national supply management system, neither refresher training nor pharmaceutical supervisory support was provided to health workers. A study exploring pharmacy-related supervision in Dodoma Region found that supportive supervision of health facilities is done without a practical and reproducible tool and indicators. Feedback and actions were rarely taken [16].

### Availability of medicines

Mean availability of 26 tracer medicines in all health facilities of Dodoma Region was 53%. The causes of stock-outs are numerous and systemic. Weaknesses of the public sector supply chain in Tanzania have been described elsewhere and relate among others to issues of funding, inefficiencies of forecasting, quantification and procurement, ordering at the level of health facilities, leakage, staff shortage and inappropriate use of medicines [32]. Stock-out rates were higher in health facilities without pharmaceutical staff than in hospitals with pharmaceutical staff. While professional competency offers a probable explanation, another interpretation could be linked to structural advantages of hospitals in terms of procedures, communication and proximity in the supply chain.

### Good storage practice

Adherence to Good storage practice guidelines was generally weak with no apparent correlation between type and staffing of a health facility. Storage practice seems to be an activity that can be managed by health workers independent of cadre and education.

### The reality of task shifting

The task of managing medicines supplies has been shifted to non-pharmaceutical staff already overburdened with clinical care and administrative activities. This informal delegation is not officially termed task shifting and happens without formal support. WHO has issued recommendations for implementing task shifting [19]. For instance, task shifting should aim at improving the overall quality of care and protect both health workers and patients. This should involve both the presence of effective regulatory mechanisms, certification and remuneration of health workers who assume new or delegated tasks. In our study, pharmaceutical task shifting was a reality for 95.5% of the surveyed health facilities albeit not formally supported. Clinical staff handling medicines supply have not been appropriately trained, are poorly supervised and not supported in their duties. Availability of medicines, the main indicator for effective supply management, is lower in health facilities without pharmaceutical staff. While this correlation may be common sense, our study does not yield any conclusion on possible causality. Task shifting is informal and unsupported by policy, regulation and capacity building which points to a gap in the practice-policy translation.

In comparison, another more formal approach of task shifting implemented in the private sector of Tanzania is the successful model of Accredited Drug Dispensing Outlets (ADDO) [34]. Nurse assistants having received one year of training after primary or secondary basic education receive formal, albeit limited ADDO training to manage drug outlets. These dispensers are accredited and recognized by the Pharmacy Council and the Tanzania Food and Drug Authority, and are supported by supervision and inspection. A study on antimicrobial use found that availability of tracer antimicrobials increased by 26%, the proportion of ADDOs with unauthorized items decreased from 53% to 13% and the percentage of ADDO dispensers following good dispensing practices increased from an average of 67% in the first monitoring visit to an average of 91% during the last visit [35].

A study in one district in Tanzania assessed potential benefits and challenges related to task shifting [36]. Issues regarding task shifting included the quality of services and the need for standardized procedures, strong supervision and mentoring. An absence of policies and regulations to guide implementation and monitoring of task shifting practice was noted. One potential issue concerned the danger that task shifting could undermine the professional distinction of formally trained professionals. The study concluded that task shifting implementation occurs as an ad hoc mechanism to cope with the existing shortages of health workers in underserved areas.

A study on perceptions and motivation regarding task shifting showed that adequate support with supportive supervision, recognition, equitable allocation of resources, training, compensation and monitoring are vital to ensure quality of care, worker morale and staff retention. In-service training and technical support were important for the success of task shifting [37].

Another study found that task shifting may be a motivational factor, especially for lower cadres [24]. Conversely, delegation of tasks perceived as less prestigious could be a disincentive. The main benefit of task shifting in relation to staff shortage was increased coverage and access to health services. Informal task shifting which happens as an ad hoc response to an observed need rather than as an explicit strategy may result in charging cadres with new, vague or overlapping responsibilities, possibly compromising quality and outcome of services such as assuring the availability of medicines. Clinicians charged with managing medicines supply are distracted from their clinical activities and may negatively influence availability of medicines due to insufficient skills, which again can lead to compromised quality of care.

## Conclusions

Task shifting and role substitution have been hailed as a promising strategy to address health worker shortages. This study has shed some light on the informally grown context in which the management of medicines is done by various cadres, particularly non-pharmaceutically trained clinical staff in Dodoma Region. Pharmacy-related tasks and supply management have been shifted to clinical health workers without formal policy guidance, explicit requirements nor guidelines, and without the necessary support through systematic training and supervision. This study concludes that informally shifting the task of medicines management to non-pharmaceutical staff may have negative implications both for availability of medicines and quality of clinical care.

A more formalized approach to task delegation is needed, including capacity building, regulatory and supervisory mechanisms and possibly remuneration, to avoid compromising access to medicines and quality of services. Implicit and informal task shifting should be recognized as a reality and formalized in order to improve outcomes. Job description, job orientation, operational procedures and simple tools may be useful to support certain tasks.

Task shifting is a reality in the pharmaceutical sector in Tanzania and its implementation occurs mainly as a coping mechanism to the existing workforce crisis rather than as a deliberate policy response to the shortage. Whether formal pharmaceutical task shifting to any duly trained cadre will lead to a better outcome in terms of availability of medicines, remains to be studied.

Further research is suggested to better understand the time spent by health workers on supply-related activities as well as to explore the perceptions and motivation of clinical staff regarding management of supply and pharmacy stores. The gap between health workers’ job descriptions and their practice should be addressed. Future research should explore optimal ways of task shifting implementation and provide guidance for evaluation of effectiveness and impact on quality and costs.

## Abbreviations

ADDO: Accredited Drug Dispensing Outlet
FIP: International Pharmaceutical Federation
GPP: Good Pharmacy Practice
GSP: Good Storage Practice
HPSS: Project, health promotion and system strengthening project
HIV: Human immunodeficiency virus
ILS: Integrated logistics system
MOHSW: Ministry of Health and Social Welfare
NEML: National Essential Medicines List
R&D: Research and development
SDC: Swiss Agency for Development and Cooperation
TB: Tuberculosis
WHO: World Health Organization
